# Obstructive Uropathy Caused by Massive Fecal Impaction in a Patient With Congenital Anorectal Malformation and Neurogenic Bladder

**DOI:** 10.7759/cureus.47036

**Published:** 2023-10-14

**Authors:** Soo Jin Lee, Seok Young Cho, Wonkyo Yi, Kyung Pyo Kang

**Affiliations:** 1 Department of Internal Medicine, Research Institute of Clinical Medicine, Jeonbuk National University Medical School, Jeonju, KOR; 2 Biomedical Research Institute, Jeonbuk National University Hospital, Jeonju, KOR

**Keywords:** urinary tract infection, fecal impaction, neurogenic bladder, anorectal malformation, obstructive uropathy

## Abstract

Anorectal malformations (ARMs) comprise a broad spectrum of congenital anomalies involving both anorectal and urogenital tracts. After diagnosis, urological problems should be evaluated in addition to surgical correction of ARMs. Commonly encountered urological problems in patients with ARMs are recurrent urinary tract infections, vesicoureteral reflux, and chronic kidney disease. Therefore, the proper timing of urination and appropriate defecation habits are essential for preserving renal function in patients with ARMs. Here, we report a case of acute hydronephrosis by severe stool impaction in a patient with a history of congenital ARMs and neurogenic bladder. In this case, the physicians should consider properly managing chronic constipation and urination in patients with ARMs despite successful surgical corrections.

## Introduction

Anorectal malformations (ARMs) consist of a broad spectrum of congenital anomalies with a reported incidence of approximately one in 5,000 live births and involve both anorectal area and urogenital tracts [1,2]. Chronic constipation is a frequently encountered functional disorder in children and adolescents after treatment of ARM [3]. The reason for chronic constipation in patients with ARMs is the lack of a normal anal canal with variable degrees of defective function of the sphincter muscle complex, usually resulting in hypomotility [4]. The incidence of constipation varies by 30~80% in ARMs, attributed to different socio-economic conditions and dietary habits among populations [4-6].

Urologic problems in patients with ARMs are also associated with significant morbidity, representing a potentially life-threatening condition, mainly severe urinary tract infection (UTI) and kidney damage [7,8]. One of the most important conditions is the neurogenic bladder associated with vesicoureteral reflux. This condition might be associated with recurrent UTI and progressive renal dysfunction, resulting in chronic kidney disease (CKD) [9].

Chronic constipation results in massive fecal impaction and causes extensive dilatation of the rectosigmoid colon, subsequently leading to compression of the ureter and bladder, which can induce post-renal acute kidney injury. Neurogenic bladder dysfunction also aggravates the dilatation of the ureter and causes hydronephrosis. Here, we report a case of acute hydronephrosis by severe stool impaction in a patient with a history of congenital ARM and neurogenic bladder.

## Case presentation

A 20-year-old woman came to the emergency department with a one-week history of persistent fever. She had a congenital ARM when she was born and underwent a revision operation right after birth. At seven, she was diagnosed with neurogenic bladder and taught to perform clean intermittent catheterization (CIC) every 4 hours. However, she had not performed CIC regularly for the last two weeks and did not use sterilized catheters. At the arrival, her blood pressure was 87/40 mmHg, pulse was 108 bpm, respiratory rate was 23 bpm, oxygen saturation was 99%, and body temperature was 37.8 ℃. 

Laboratory examination showed the following results: white blood cell count of 22,700/mm^3^, hemoglobin of 7.9 g/dL, platelet of 229,000/mm^3^, blood urea nitrogen (BUN) level of 84 mg/dL, serum creatinine level of 3.24 mg/dL, and C-reactive protein (CRP) level of 102.51 mg/dL. Arterial blood gas analysis showed pH 7.176, HCO3- 5.6 mmol/L, pCO_2_ 15.2 mmHg, and lactate 1.0 mmol/L. Urinalysis revealed 2+ proteinuria, microscopic hematuria, and pyuria. However, there was no growth in either blood or urine cultures. 

**Figure 1:**
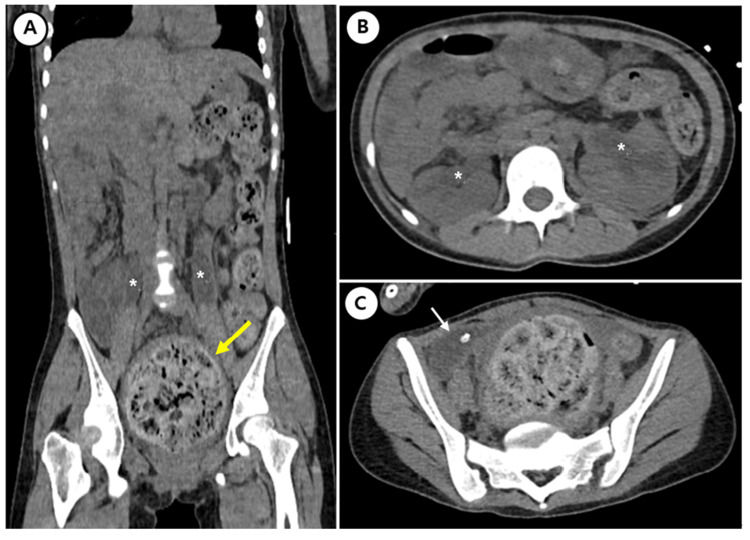
Non-enhanced computed tomography of the abdomen (A) Computed tomography of the abdomen (coronal plane) reveals a large amount of stool retention (yellow arrow) and its upstream dilated distal ureter (*). (B) Computed tomography of the abdomen (axial plane) shows both hydronephroses (*). (C) Computed tomography of the abdomen (axial plane) indicates a collapsed bladder due to a distended rectum (white arrow).

After the initial laboratory assessment, Foley catheterization was undertaken. However, the drained urine amount was 90 mL immediately after Foley catheterization. Next, we ordered non-enhanced computed tomography (CT) to assess the post-renal cause of acute kidney injury. CT of the abdomen revealed a dilated rectum with impacted feces, with the largest diameter of the rectum measuring 10.2 cm. Hydroureteronephrosis was also present due to compression of the distal ureter secondary to fecal retention (Figure 1).

After receiving a glycerin enema three times and continual use of oral laxatives, she defecated more than twice per day and maintained a urine output of over 100 mL/h. Subsequently, laboratory results showed marked improvement in metabolic acidosis (bicarbonate of 21.9 mmol/L) and renal function (BUN 50 mg/dL, serum creatinine 1.23 mg/dL). After six months, follow-up renal ultrasonography showed that despite improving both hydronephroses, uneven caliectasis remained in both kidneys.

## Discussion

Constipation is a common problem after the surgical treatment of ARMs [4]. Failure to treat it adequately can cause morbidities such as fecal impaction, overflow pseudo incontinence, megacolon, and urologic problems [10]. Fecal impaction and hypomotility dilate the rectum over time and worsen constipation, creating a vicious cycle [4]. Dilatation of the rectosigmoid colon might also compress the urinary tract, leading to obstructive uropathy [11]. A fiber diet and laxatives are the first treatments to provide adequate bowel movements for emptying the bowel. However, caution should be prescribed in patients with ARMs because stool softeners can cause fecal incontinence [12]. Therefore, early recognition of problems associated with constipation is essential to provide appropriate medical treatment to avoid constipation-related morbidities after surgery of ARMs.

About 30~50% of all patients with ARMs are associated with genitourinary defects such as vesicoureteral reflux (VUR) or neurogenic voiding dysfunction [9,13,14]. It can cause serious problems affecting other organs, especially the kidneys. Therefore, all patients should be screened at birth to rule out these defects [1]. For early diagnosis, it is crucial to suspect, diagnose, and treat urogenital abnormalities properly, and treatment will prevent possible parenchymal damage [8,14]. This patient was diagnosed with a neurogenic bladder relatively later, at the age of seven years. Urologic evaluation prior to colostomy might provide a pediatric surgeon with the information to address the urologic problem at the time of the colostomy [1]. This patient might have the first experience with urosepsis due to obstructive uropathy associated with severe fecal impaction. Despite the recovery of renal function, follow-up ultrasonography shows uneven caliectasis in both kidneys, which may progress to CKD in the future. The progression to end-stage renal disease in patients with ARMs is associated with neurogenic bladder, VUR, and recurrent UTI [9]. Therefore, controlling intravesical pressure and preventing recurrent UTIs is essential. CIC and bladder augmentation showed a definitive positive effect in protecting renal functions in patients with neurogenic bladder [8]. Also, patients with ARM should be followed for life, monitoring their kidney function and the anatomy of the urinary tract, since its complications may take 10-20 years to develop [14]. In this case, after a diagnosis of neurogenic bladder, this patient has performed regular intermittent catheterization, which is sometimes missed. Therefore, patients’ and their parents' education about performing CIC is critical for preventing recurrent UTI and the progression of renal dysfunction.

## Conclusions

In conclusion, physicians should consider that severe stool impaction may be a possible cause of acute hydronephrosis and underlying the fact that the proper timing of urination and the appropriate defecation habits are essential for preserving renal function in patients with ARMs. Furthermore, constipation negatively affects these patients' quality of life, but it is usually not life-threatening. However, since urologic problems are considered a critical source of morbidity, one of the goals in managing patients with ARMs should be preserving renal function.
